# A Novel Graphene-Based Nanomaterial for the Development of a Pelvic Implant to Treat Pelvic Organ Prolapse

**DOI:** 10.3390/jfb15110351

**Published:** 2024-11-20

**Authors:** Amelia Seifalian, Alex Digesu, Vik Khullar

**Affiliations:** Department of Urogynaecology, Imperial College, London W2 1NY, UK; a.digesu@imperial.ac.uk (A.D.); vik.khullar@imperial.ac.uk (V.K.)

**Keywords:** graphene, regenerative medicine, nanotechnology, gynaecology, nanomaterials, Hastalex

## Abstract

Graphene is the wonder material of the 21st century, promising cutting-edge advancements in material science with significant applications across all industries. This study investigates the use of a graphene-based nanomaterials (GBNs) ans trade-registered Hastalex^®^, as novel materials for surgical implants aimed at treating pelvic organ prolapse (POP). This study investigates the mechanical properties and physicochemical characteristics of the material, mainly focusing on its potential to address the limitations of existing polypropylene (PP) implants, which has been associated with numerous complications and banned across multiple countries. Attenuated total reflectance Fourier transform infrared (ATR-FTIR) confirmed the bonding between functionalised graphene oxide (FGO) and the base polymer chain. Hastalex exhibited excellent mechanical properties with 58 N/mm^2^ maximum tensile strength at break and 701% elongation at break, whilst maintaining its shape with no plastic deformation. These results were comparable to that of sheep pelvic muscular tissue. Hastalex demonstrated its hydrophilic properties from contact angle measurements. Scanning electron microscopy (SEM) and atomic force microscopy (AFM) showed a uniform plane with surface nanotopography, promoting cell-to-material interaction. The results confirmed the suitability of Hastalex in the development of a new pelvic membrane to treat POP.

## 1. Introduction

**Figure 1:**
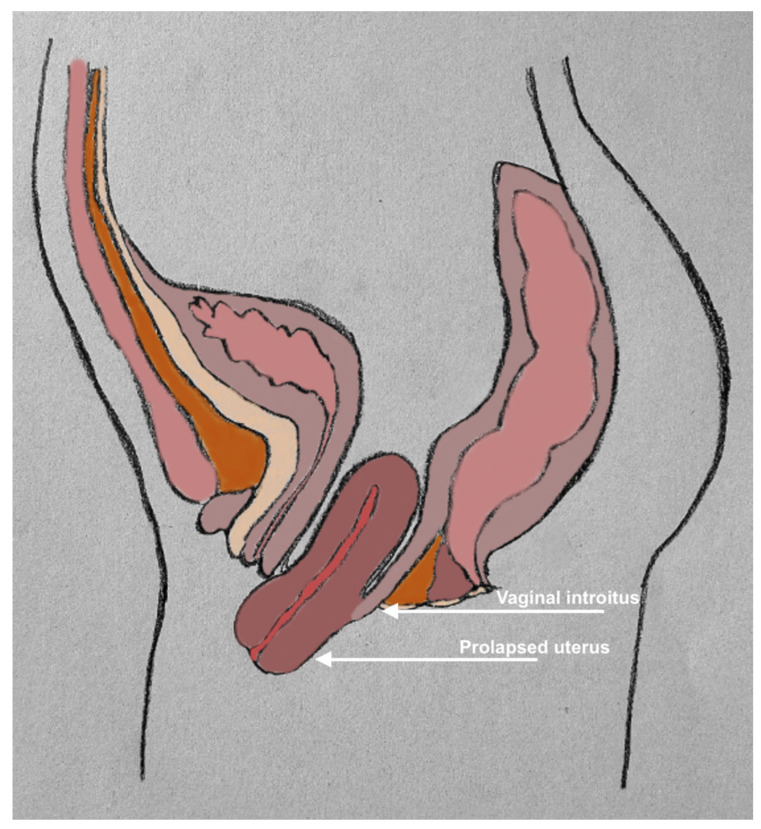
Schematic diagram demonstrating pelvic organ prolapse.

Pelvic organ prolapse (POP) is a benign condition characterised by the descent of pelvic organs down the vaginal canal due to the weakening of the pelvic floor and soft tissue structures as seen in Figure 1 [1]. Descending organs can include the bladder, rectum, or/and uterus; and thus, cause symptoms of pain, discomfort, bulging sensations, urinary retention and incontinence, and faecal incontinence. Risk factors include age and obesity, both of which are on the upward trend and therefore increasing the prevalence of POP. Initial conservative management involves watchful waiting, or the use of a silicone pessary, which serves as a temporary scaffold to support prolapsed organs. However, should these methods prove inadequate, then invasive treatment with surgical intervention is required.

Surgical intervention for the treatment of POP can include native tissue repair, but a five-year follow-up shows failure rates of over 60% [2]. The use of an adjunct was implemented to improve surgical outcomes and prevent the recurrence of POP. Initially, biologic implants of autologous fascia, allografts, or xenografts, were used, but synthetic implants rose to popularity due to being cheap and readily available and were considered to have better outcomes. Polypropylene (PP) mesh became the most popular, inserted trans-abdominally or transvaginally. This mesh has since been banned in a number of countries including UK, USA, Canada and several others, due to concerns over the safety of the material. A number of complications occurred with the PP mesh, including mesh exposure, chronic infection, and chronic pain, secondary to the mismatch of the properties between PP and native pelvic tissue [3]. For the future investigation of new pelvic implants, sheep have demonstrated similar pelvic anatomy and are cheap and readily available, hence most suitable for preclinical animal studies [4].

Currently, there is an unmet clinical need for the treatment of POP. Surgeons and their patients are desperately seeking non-toxic and biocompatible material with similar viscoelastic properties to the surrounding pelvic tissue to address the needs of this condition. 

Graphene may just be the perfect material for such an application. Graphene is a two-dimensional monolayer of carbon atoms tightly bound in a hexagonal honeycomb lattice. Graphene has excellent mechanical strength, 200× stronger than steel, whilst being highly elastic and lightweight [5]. Graphene has been labelled as the wonder material of the 21st century, with its properties being harnessed as graphene-based nanomaterials (GBNs) [6]. GBNs have been emerging in every industry, from aerospace, electronics, energy storage and batteries, textiles and wearable technology, sensors, and biomedical and healthcare applications [7]. 

We have developed and patented a technology to manufacture a new breed of novel nanocomposite material for biomedical application. This material is based on functionalised graphene oxide (FGO) and has been trade registered as Hastalex^®^. The enhanced biocompatibility, superior mechanical engineering properties, and augmented degradative resistance of Hastalex^®^ render it capable of functioning as a scaffold for bioartificial organs [8], under development for the heart valve [9], as conductive nanofibres for nerve regeneration [10], and for wound healing purpose [11]. The application of Hastalex in pelvic implants presents a groundbreaking opportunity to improve patient outcomes by addressing the limitations of current PP implants.

The aim of this study was to evaluate Hastalex for the development of a new pelvic membrane to augment POP surgery. Its promising outlook for the treatment of POP was confirmed with the characterisation of the physicochemical properties of the material, offering a potential long-term cure to the condition, which is currently an unmet clinical need.

## 2. Materials and Methods

### 2.1. Materials

The materials included in this study were Hastalex^®^, poly(carbonate-urea)urethane (PCU), functionalised graphene oxide (FGO) powder (NanoRegMed Ltd., London, UK), polypropylene (PP) (RS Components Ltd., Northants, UK), sheep pelvic muscle tissue (obtained from abattoir, London, UK), and graphene oxide (GO) powder (Sigma Aldrich Ltd., Gillingham, UK). 

### 2.2. Hastalex Polymer

Hastalex is an advanced biocompatible material composed of FGO nanoparticles covalently attached to the PCU backbone [12]. Hastalex is a highly durable and biostable material which has been proven to be non-toxic and biocompatible [13]. It is non-biodegradable, and thus suitable for permanent surgical implants. The use of PCU sheets was to demonstrate the significance of the physiochemical improvement of Hastalex by covalently conjugating FGO on the PCU polymer chain at pre-polymer synthesis.

### 2.3. Preparation of Materials for Investigation

A solution of Hastalex polymer was poured evenly onto a glass template and placed in the oven at 60 °C for twelve hours. Sheets of Hastalex material were then cut into shapes as appropriate and described for each of the investigations, thus providing Hastalex sheets via dry casting. The PCU polymer was similarly poured evenly onto a glass template and placed into the oven at 60 °C for twelve hours. This provided sheets of the material ready for investigations. 

It is important to mention that when Hastalex was placed in the oven, one side would be in contact with the glass tray, with the opposite side in contact with air. Hence, the Hastalex sheets would demonstrate a shiny side, in contact with the glass, and an opaque side, in contact with the air. 

### 2.4. Surface Chemistry Structure Using ATR-FTIR

The surface chemical functional groups were investigated using attenuated total reflectance Fourier transform infrared (ATR-FTIR) (Nicolet Summit X FTIR Spectrometer, Thermo Fisher Scientific, Franklin, MA, USA). Infrared was directed at the sample and absorbed at different wavelengths depending on the surface functional groups of the material. The samples that were included were GO powder, FGO powder, and a Hastalex sheet. 

The resulting spectrum were analysed for each sample using the OMNIC paradigm software (Thermo Fisher Scientific, Franklin, MA, USA). A total of 16 scans were performed for each sample at 400 to 4000 cm^−1^ and the mean was plotted on to a spectrum graph.

### 2.5. Mechanical Properties

#### 2.5.1. Uniaxial Tensile Test

The mechanical properties of all the materials, Hastalex, PP, PCU, and the sheep muscle samples, were investigated via uniaxial tensile testing. A tensile tester (AML Instruments Ltd., Lincoln, UK) was equipped with the appropriate software (THSSD-2018) and used to characterise the mechanical properties of the aforementioned materials (*n* = 4 for all materials). The samples were cut into dog bone shapes with the main body dimensions of 4 mm × 30 mm, as per the ISO 527 [14]. 

The experiment was performed under uniaxial tension at 50 mm/min. Data were analysed to calculate the tensile strength at the 100% strain, the 200% strain, the 300% strain, the maximum tensile strength at break, and the elongation at break. Young’s modulus was extrapolated from the stress–strain curves. 

#### 2.5.2. Elastic Test of Deformation

The samples of Hastalex, PP, PCU, and sheep muscle were subject to 50% stretch for 40 cycles and investigated for deformation following completion. The samples were cut into a dog bone shape as per aforementioned ISO standards and underwent stretch as described. The samples were then removed from the instrument and photographed to depict the changes in the shape. 

### 2.6. Assessment of Hydrophilicity of the Material

The hydrophobicity of the PP, PCU, and Hastalex samples were investigated by testing water adsorption on the surface of the samples using the appropriate instrument, Attension Theta (Biolin Scientific, Gothenburg, Sweden). A sessile drop analyser was used for the contact angle measurements (*n* = 3). The measurements were taken in an air-conditioned laboratory at 25 °C.

A drop shape analysis was performed using the OneAttension software (Biolin Scientific, Gothenburg, Sweden). The angles >90° were considered hydrophobic and the angles <90° were considered hydrophilic.

### 2.7. Assessment of Surface Topography

#### 2.7.1. Scanning Electron Microscope (SEM)

The surface topography and cross-sections of the material were investigated using SEM. The samples of Hastalex were prepared by cutting 10 mm × 10 mm squares. These samples were then attached to aluminium stubs using double-sided carbon sticky tape and underwent 15 nm chromium coating (Q150T, Quorum Ltd., East Sussex, UK). An analysis was then performed with SEM (LEO 1525, Zeiss, Oberkochen, Germany) and the samples were observed at ×500, ×1000, and ×5000 magnifications at 5 kV.

#### 2.7.2. Atomic Force Microscope (AFM)

The nanosurface topography of the PCU and the Hastalex was measured with AFM using the MFP-3D Origin AFM Asylum Research (Oxford Instruments, Abingdon, UK). Flat sheets of PCU and Hastalex were prepared into 10 mm × 10 mm squares. AFM was performed using the tapping mode with the SCOUT-150 probe (NuNano, Bristol, UK) (*n* = 3). Data were processed to analyse the surface topography and roughness of the samples.

### 2.8. Statistical Analysis

Significance was determined by performing a one-way analysis of variance (ANOVA) with Turkey’s multiple comparison post hoc tests. A statistical analysis was performed using GraphPad Prism 6 (GraphPad software, San Diego, CA, USA). A *p* value of less than 0.05 was considered significant. A statistical analysis was applied to the uniaxial tensile tests of the mechanical properties of the materials and the contact angle measurements.

## 3. Results

### 3.1. Surface Chemistry Structure Using ATR-FTIR

**Figure 2:**
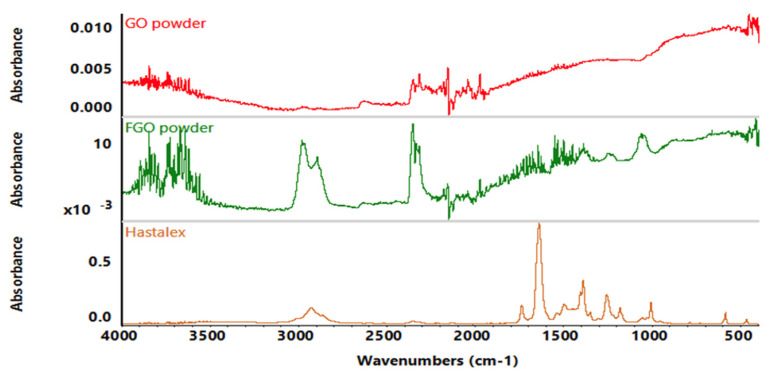
ATR-FTIR spectrum for GO and FGO powder and Hastalex. Spectrum analysis performed and peak 3000 cm^−1^ representing amine group functionalisation.

ATR-FTIR was used to analyse the surface chemical changes. Amine group functionalisation was confirmed in the FGO powder and the Hastalex. The GO powder was used as a control group in this instance. See the amine peak and the structure of the spectrums analysed in Figure 2.

### 3.2. Mechanical Properties

#### 3.2.1. Uniaxial Tensile Test

**Figure 3:**
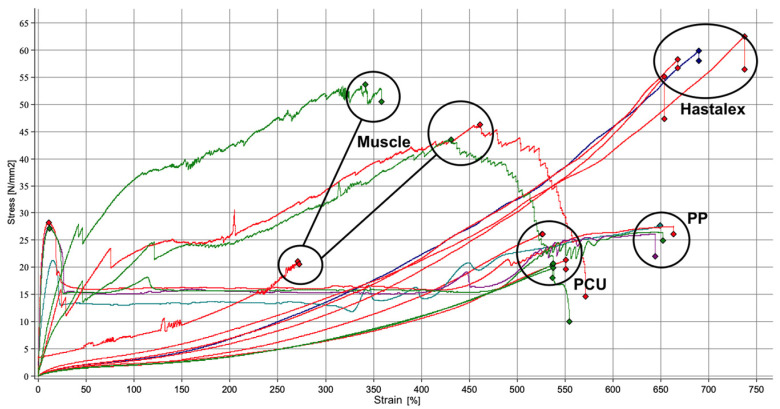
Tensile stress–strain graph of data from the tensile test on Hastalex; muscle tissue; PCU, poly(carbonate-urea)urethane; and PP, polypropylene.

The raw mechanical properties of the materials were demonstrated on the stress–strain curve shown in Figure 3 and summarised in Table 1. Statistical analysis confirmed that Hastalex was significantly stronger (*p* < 0.001) than both the PP and the PCU, but not significantly stronger than muscle tissue. The elongation at break for the Hastalex, the PP, and the muscle was similar amongst the three groups, but all significantly greater than that of the PCU. The Hastalex elongation at break was 701% (mean) versus the PCU being 532% (mean), demonstrating a statistically significant difference of *p* < 0.001.

#### 3.2.2. Elastic Test of Deformation

**Figure 4:**
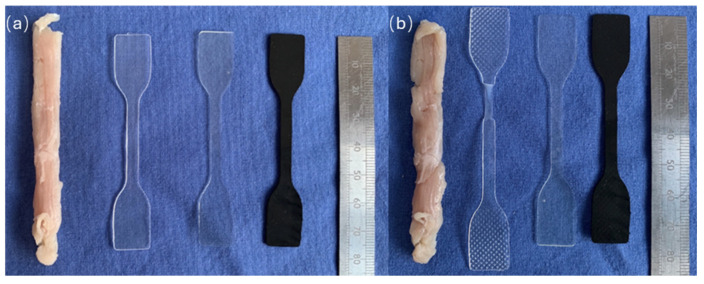
Assessment of PP, PCU, Hastalex, and sheep pelvic tissue for their mechanical properties (**a**) left before and (**b**) right after subjection to a 50% stretch for 40 cycles.

The deformation of the samples were considered as any change in the shape following the stretch cycles. The PP was unable to retain its shape and underwent plastic deformation, which was evident from the visual analysis. All the other materials macroscopically maintained their shape. See Figure 4a,b for before and after images of the investigations, respectively.

### 3.3. Assessment of Hydrophilicity of the Material

**Figure 5:**
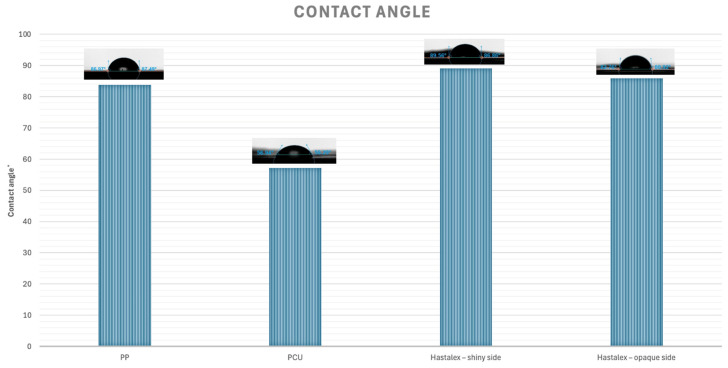
Bar chart demonstrating contact angles of materials under investigation. Contact angles were PP (83.8 ± 2.4°); PCU (57.2 ± 0.4°); Hastalex (shiny side 89.1 ± 0.9°; opaque side 85.9 ± 0.7°).

Hastalex, PP, and PCU were all categorised as hydrophilic with contact angles <90 °C. The results demonstrated that PP (83.8 ± 2.4°) and Hastalex (shiny side −89.1 ± 0.9°; opaque side −85.9 ± 0.7°) had similar hydrophilicity but were both significantly different to the more hydrophilic PCU (57.2 ± 0.4°) with *p* < 0.001. All the results were mean values ± standard error of the mean. These results have been demonstrated on a bar chart, as seen in Figure 5.

### 3.4. Assessment of Surface Topography

#### 3.4.1. Scanning Electron Microscope (SEM)

**Figure 6:**
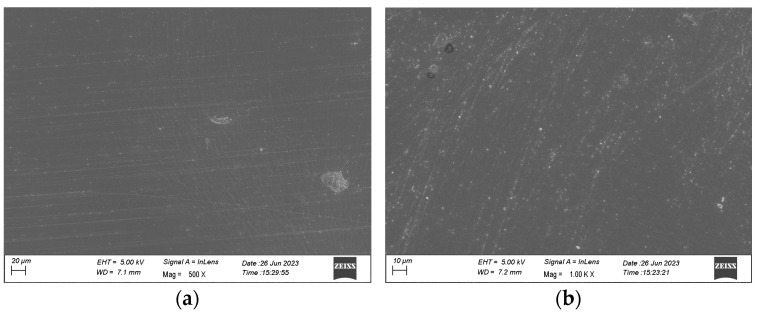
SEM images of the Hastalex sheet; (**a**) side of sheet in contact with glass during casting; and (**b**) side of sheet in contact with air during casting.

The surface topography of the samples was investigated using SEM, as described. The shiny side is seen in Figure 6a and the opaque side in Figure 6b. As expected, the opaque side shows more surface markings; however, both sides show a uniform surface with regular surface markings.

#### 3.4.2. Atomic Force Microscope (AFM)

**Figure 7:**
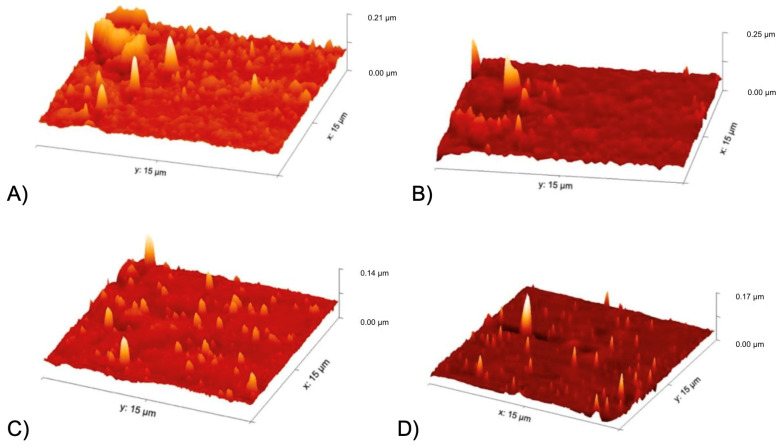
AFM images capturing surface nanotopography of AFM images of (**A**) PCU side in contact with glass during casting; (**B**) PCU side in contact with air during casting; (**C**) Hastalex side in contact with glass during casting; (**D**) Hastalex side in contact with air during casting.

The samples were analysed using AFM as per the methodology above. See the results in Figure 7. The opaque side of the material, in contact with air, did demonstrate greater roughness in comparison to the shiny side, in contact with the glass. The PCU had roughness with a peak reaching 0.21 µm and 0.25 µm on the shiny and opaque sides, respectively. This is compared to Hastalex with peaks of 0.14 µm and 0.17 µm on the shiny and opaque sides, respectively.

## 4. Discussion

The significance of the GBNs in the application of tissue implant for regenerative medicine comes with its excellent mechanical properties. For the success of a surgical adjunct in the treatment of POP, the implant must mimic the mechanical properties of a healthy pelvic floor tissue—strong yet elastic [15]. POP effects the smooth muscle tissue of the vagina, resulting in its stiffness and rigidity [16]. GBN presents enhanced cell interaction, hence better cell adhesion, differentiation and proliferation on the material surface [17]. Graphene has also demonstrated anti-bacterial properties which can prevent chronic infection following POP surgery [18]. The anti-bacterial mechanism of action has been derived from its surface topography and wettability [19]. 

There are multiple types of surgeries to treat POP depending on the type and location of the prolapse. The initial surgery that the new pelvic membrane would be applied to is planned to be sacrocolpopexy. Sacrocolpopexy is the common gold-standard procedure used for apical prolapse [20], with a well-established standardised technique ensuring reliable outcomes. The properties of Hastalex will benefit sacrocolpopexy surgery with better biocompatibility and less post-operative complication compared to the PP mesh, as previously described. With further development, the aim is to apply the implant to all POP procedures and, following this, to stress urinary incontinence procedures also.

Currently, Hastalex is manufactured at NanoRegMed, London UK, under collaboration with the authors of the paper. Regenerative medicines often carry higher initial production costs; however, as the product is further developed and produced on a large-scale basis, the costs decrease. The price of the Hastalex pelvic membrane is expected to be competitive with that of the PP mesh, and the simultaneous lack of long-term complication will further improve its cost effectiveness as a product to treat POP. 

The amine functionalisation of FGO allows it to form strong covalent bonds with the base polymer, PCU, and enhance the properties of the material. The PCU base polymer has been used in other compounds with enhanced tissue integration response [21]. ATR-FTIR is used to analyse the surface functional groups of the sample being investigated. ATR-FTIR has application in understanding the composition of small samples, particularly in quality control, or checking for the success of a reaction. In this experiment, ATR-FTIR was used to detect the amine group of FGO, with further analysis to confirm FGO incorporation in Hastalex. 

The Hastalex samples were shown to have similar a Young’s modulus to the sheep pelvic tissue analysed in the mechanical properties’ investigations. Sheep pelvic tissue was used due to its similarity to human pelvic tissue, as previously reviewed [22]. This was in addition to a similar maximum tensile strength. The results of the mechanical investigations demonstrated greater tensile strength of Hastalex compared to PP. Investigation of human pelvic tissue’s mechanical properties in vivo presented difficulties, and those on cadaveric structures were less accurate due to structural loss once explanted [23]. A previous investigation of prolapse vaginal tissue found similar results to the Hastalex and sheep pelvic tissue on the stress–strain curve in regard to elongation and tensile at break [24]. 

Investigation of the mechanical properties included sheep muscle tissue as a comparator for mammalian pelvic floor. However, sheep tissue proved extremely difficult to include in the above investigations due to being cadaveric and falling apart easily. There were also extensive difficulties in shaping the muscle according to the dog bone shape used for consistency. In addition, the direction of muscle fibres was given extra attention to ensure maximal accuracy of the results. 

The elasticity of the materials was also investigated and captured. The most essential finding was that the PCU, Hastalex, and sheep pelvic tissue exhibited resilience and returned to their original state after the stretch cycles. In contrast, PP, being a thermoplastic material, experienced plastic deformation and permanent change after the initial stretch and did not recover. Mesh deformation is a prominent cause of severe complications in POP surgery [25]. A new implant for this application would need to be durable and maintain its shape in the environment of the pelvic floor. The pelvic floor undergoes frequent cycles of stretch secondary to the changes in pressure, from variations in the posture, exercise, and activity (e.g., coughing, sneezing) [26]. The GBN was stretchable and was hence found suitable for implantation in the pelvic floor [27]. 

Wettability experiments were performed using contact angle analysis and the results confirmed the moderately hydrophilic surface of the Hastalex. Hydrophilic materials enhance cell adhesion and proliferation [28]. Moderate hydrophilicity has demonstrated better cell adhesion and interaction compared to superhydrophilic or hydrophobic surfaces, additionally preventing bacterial adhesion [29]. 

There are multiple proposed mechanisms for the enhanced cellular outcomes of hydrophilic surfaces. One proposed mechanism is that greater wettability distributes biological fluids more evenly across the material surface, allowing cells to come into close contact with the material, hence enhancing the cell-to-material interaction [30]. Additionally, the biological fluid/blood surface spread on the surface improves protein layer formation, as key components of the extracellular matrix adsorb more readily on the hydrophilic surface [31]. The protein layer exposes the binding sites favourable for stronger and more specific cell attachment [32]. GO has typically demonstrated properties of an effective and stable additive that improves surface hydrophilicity [33]. 

Surface topography is essential to tissue integration of any surgical graft and surface modifications can have large impacts on cell attachment, differentiation, and proliferation behaviour [34]. The SEM and AFM performed on the Hastalex demonstrated uniform surface topography. Surface nanotopography enhances cell-to-material interaction by increasing the surface area available for cell adhesion and structurally imitating the extracellular matrix [35]. The AFM analysis of the Hastalex showed surface nanotopography with surface roughness and evenly distributed peaks.

While this study demonstrates promising results with Hastalex, further in vivo studies are necessary to fully understand its biocompatibility and long-term performance in a dynamic physiological environment. Future research should explore the degradation behaviour of Hastalex in different biological conditions to ensure its stability and safety for long-term implantation.

## 5. Conclusions

Hastalex has perversely been shown to be biocompatible [13] and non-toxic, according to the unpublished data tested by Pharmidex Ltd., London, UK. Here, we have characterised the physicochemical properties of Hastalex. The results demonstrate that Hastalex consistently out-performed the other synthetic materials under investigation in these experiments. Thus, Hastalex has shown its potential for development as a membrane for the treatment of pelvic organ prolapse. The work is in progress for the design of a pelvic membrane using Hastalex and for preclinical studies using sheep as the animal model, which has demonstrated similar anatomy to human pelvic tissue [4]. 

## Figures and Tables

**Table 1:** Mechanical properties following the analysis of PP, PCU, Hastalex, and muscle. The table shows the average of the data calculated at each point ± the standard error of the mean.

Material	Tensile at 100% Strain (N/mm^2^)	Tensile at 200% Strain (N/mm^2^)	Tensile at 300% Strain (N/mm^2^)	Maximum Tensile Strength at Break (N/mm^2^)	Elongation at Break (%)	Young’s Modulus (kPa)
Hastalex	3.3 ± 0.3	7.0 ± 0.5	13.0 ± 0.6	58.0 ± 1.0	701.0 ± 14.8	78.5 ± 12.0
PP	15.3 ± 0.8	15.3 ± 0.5	15.1 ± 0.7	25.2 ± 1.2	652.0 ± 4.0	5011.4 ± 343.9
PCU	2.4 ± 0.3	4.4 ± 0.5	8.1 ± 1.0	23.3 ± 1.9	531.8 ± 7.9	71.3 ± 8.6
Muscle	21.8 ± 5.9	26.3 ± 6.1	39.2 ± 6.3	40.6 ± 6.8	392.5 ± 48.1	728.0 ± 226.2

## Data Availability

The original contributions presented in this study are included in the article; further inquiries can be directed to the corresponding author.
