# Correction: Seifalian et al. A Novel Graphene-Based Nanomaterial for the Development of a Pelvic Implant to Treat Pelvic Organ Prolapse. *J. Funct. Biomater.* 2024, *15*, 351

**DOI:** 10.3390/jfb16070239

**Published:** 2025-06-30

**Authors:** Amelia Seifalian, Alex Digesu, Vik Khullar

**Affiliations:** Department of Urogynaecology, Imperial College, London W2 1NY, UK; a.digesu@imperial.ac.uk (A.D.); vik.khullar@imperial.ac.uk (V.K.)

## Missing Conflicts of Interest

In the original publication [1], the author did not fully declare the Conflicts of Interest. The author has updated the Conflicts of Interest statement as follows:

**Conflicts of Interest:** Amelia Seifalian is a shareholder of the company NanoRegMed Ltd. and her father is a director of the company NanoRegMed Ltd. The other authors declare no conflicts of interest, including any potential commercial interests.

The authors state that the scientific conclusions are unaffected. This correction was approved by the Academic Editor. The original publication has also been updated.
